# Pembrolizumab-Associated Polyserositis with Eosinophilic Pleural Effusion During Adjuvant Therapy for Clear Cell Renal Cell Carcinoma: A Case Report and Targeted Review

**DOI:** 10.3390/curroncol33060314

**Published:** 2026-05-27

**Authors:** Mikel Portu, Judit Sanz-Beltran, María Alejandra Duarte Borges, Julieta Navarro, Alexandra Arias, Paula Alvarez, Angel Fernández-Rebollo, Carlos Reyes, Juan Flores, Georgia Anguera, Pablo Maroto

**Affiliations:** Department of Medical Oncology, Hospital de la Santa Creu i Sant Pau, 08041 Barcelona, Spain

**Keywords:** pembrolizumab, renal cell carcinoma, polyserositis, eosinophilic pleural effusion, case report

## Abstract

Pembrolizumab is standard adjuvant therapy for high-risk clear cell renal cell carcinoma, but serositis is an uncommon immune-related adverse event that may mimic recurrence or infection. We report a 55-year-old man who achieved no evidence of disease after nephrectomy and metastasectomy and developed anasarca, large bilateral pleural effusions, mild ascites, peripheral eosinophilia, and a small pericardial effusion after six cycles of adjuvant pembrolizumab. Pleural fluid was exudative and contained 20% eosinophils. Cytology showed inflammatory cells without evidence of malignancy; bacterial, mycobacterial, and fungal studies were negative; and mildly elevated adenosine deaminase did not support tuberculosis. Cardiac function and natriuretic peptides were preserved. Pembrolizumab was discontinued, thoracentesis and corticosteroids were administered, and symptoms, eosinophilia, renal function, and albumin improved rapidly. Follow-up through March 2026 showed no oncologic progression, although some residual pleural and abdominal fluid persisted alongside imaging findings suggestive of portal-hypertension physiology, which may have contributed to residual fluid but did not explain the eosinophilic pleural syndrome. In a targeted literature review, effusion eosinophil data were infrequently reported. This case highlights a likely underrecognized eosinophilic pleural-fluid phenotype within pembrolizumab-associated polyserositis and supports routine differential cell counts in drained serosal fluid when immune-related serositis is suspected.

## Simple Summary

Immune checkpoint inhibitor therapy for kidney cancer can rarely cause inflammation of the pleural, peritoneal, and pericardial linings, which may mimic infection or tumor recurrence. We report a man receiving adjuvant pembrolizumab for clear cell renal cell carcinoma who developed edema, large bilateral pleural effusions, mild ascites, peripheral blood eosinophilia, and a small pericardial effusion. Pleural fluid contained 20% eosinophils, while cytology and microbiologic studies were negative. He improved after pembrolizumab discontinuation, thoracentesis, and corticosteroids and remained free of cancer progression during follow-up. In our targeted review of published reports of immune checkpoint inhibitor-associated serositis, effusion eosinophil percentages were seldom reported. Routine differential cell counts in drained serosal fluid may help oncologists recognize an underreported eosinophil-rich pleural fluid pattern and distinguish immune-related toxicity from infection or recurrence.

## 1. Introduction

Pembrolizumab improves disease-free survival and, with longer follow-up, overall survival as adjuvant therapy for patients with high-risk clear cell renal cell carcinoma after nephrectomy [1,2]. The KEYNOTE-564 risk framework now guides much of this practice, including treatment of selected patients rendered disease-free after complete resection of metastatic sites (M1 no evidence of disease, M1-NED) [1,3]. However, the KEYNOTE-564 M1-NED subgroup was narrowly defined, requiring complete resection of the primary tumor and soft-tissue metastases within one year of nephrectomy. Patients who deviate from these criteria should therefore be distinguished from the directly trial-defined population when applying adjuvant pembrolizumab. As the use of immune checkpoint inhibitors expands across disease settings, immune-related adverse events increasingly influence treatment delivery and survivorship in oncology practice [4,5,6].

Serosal inflammation under immune checkpoint inhibitor therapy is uncommon but increasingly documented. Reported phenotypes include pleural effusions, pericardial disease, ascites, and multi-compartment polyserositis, often presenting as dyspnea, edema, or apparent radiologic progression [7,8,9,10,11,12,13,14]. Because these presentations can mimic malignant relapse, infection, heart failure, or other systemic causes of fluid accumulation, diagnosis is often delayed, and management can be challenging [4,5,6,12,14].

Eosinophilia is also a recognized class effect of programmed cell death 1/programmed death ligand 1 blockade [15,16,17]. Pleural fluid differentials, including eosinophil percentages, can help classify inflammatory effusions [18,19,20,21]. We report a case of probable pembrolizumab-associated polyserositis with eosinophilic pleural effusion during adjuvant therapy for clear cell renal cell carcinoma and summarize a targeted review focusing on the reporting of serosal fluid differentials and effusion eosinophil data.

## 2. Detailed Case Description

A 55-year-old man with Wolff-Parkinson-White syndrome previously treated with ablation and active tobacco use underwent right nephrectomy and adrenalectomy for clear cell renal cell carcinoma on 21 May 2024 (pT1b, grade 2). He later developed a solitary osseous metastasis to the left proximal humerus, which was completely resected with limb-sparing surgery and megaprosthesis reconstruction on 30 May 2025, with negative margins. Restaging computed tomography on 3 July 2025 showed no evidence of disease, thereby achieving metastatic disease with no evidence of disease (M1-NED) status. Pembrolizumab 200 mg every 3 weeks was initiated on 15 July 2025 using the KEYNOTE-564 dose and schedule (Table 1). The interval from nephrectomy to metastasectomy was 374 days (12 months and 9 days). Restaging was performed 34 days after metastasectomy, and pembrolizumab was initiated 46 days after metastasectomy and 12 days after restaging. These details are relevant because the trial-defined M1-NED subgroup focused on soft-tissue metastases resected within one year of nephrectomy. Thus, although the KEYNOTE-564 regimen was used, this case did not fully match the published trial-defined M1-NED subgroup [1,3].

After six cycles (approximately 18 weeks), he developed progressive scrotal and lower abdominal edema with exertional dyspnea. Pembrolizumab cycle 7 had initially been withheld after a local diagnosis of presumed acute orchitis based on scrotal pain and swelling, but symptoms did not resolve as an isolated infectious process despite antibiotics. Subsequent evaluation showed bilateral scrotal edema/hydrocele together with facial/pelvic edema, pleural effusions, ascites, and peripheral eosinophilia. The episode was therefore interpreted retrospectively as an early manifestation of the evolving anasarca/polyserositis syndrome rather than proven isolated infectious orchitis. An isolated immune-mediated orchitis phenotype was considered less likely, because the scrotal findings evolved together with generalized edema and multi-compartment serosal fluid. Computed tomography showed large bilateral pleural effusions and mild ascites without evidence of tumor progression (Figure 1). He was admitted shortly thereafter for worsening anasarca.

Peripheral eosinophilia had already been documented before overt serositis (absolute eosinophil count 1.30 × 10^9^/L on 18 November 2025) and persisted at presentation (1.27 × 10^9^/L; 15.6%). Laboratory testing also showed hypoalbuminemia (33.6 g/L), mildly impaired renal function (creatinine 133 μmol/L, estimated glomerular filtration rate 51.5 mL/min/1.73 m^2^), and only mildly elevated C-reactive protein (7.9 mg/L).

Therapeutic thoracentesis removed 1.5 L of serous fluid. The pleural fluid was exudative by Light’s criteria (protein criterion met) [22] and contained 20% eosinophils, consistent with eosinophilic pleural effusion (≥10%) [18,19]. Detailed pleural fluid and paired serum data are provided in Table 2. Cytology showed reactive mesothelial cells and an inflammatory population with lymphocytes, eosinophils, histiocytes, and mesothelial cells and was negative for malignancy. Bacterial, mycobacterial, and fungal studies were negative; the pleural culture was received on 15 December 2025 and finalized as negative on 4 February 2026. Adenosine deaminase was mildly elevated at 42.8 U/L (upper limit of normal, 40 U/L); so, tuberculous pleuritis was specifically considered. However, pleural fluid mycobacterial studies were negative, and no additional clinical or microbiologic findings supported tuberculosis. Pleural fluid pH, triglycerides, and cholesterol were not available for the December index sample. Ascitic fluid was not sampled during the December admission, because ascites was mild, and the dominant symptomatic and accessible compartment was pleural.

Transthoracic echocardiography was technically limited by a poor acoustic window but showed a nondilated left ventricle with preserved systolic function (left ventricular ejection fraction 71% by Teichholz), visually nondilated atria, visually preserved right ventricular size and function, no significant mitral or aortic valve abnormality on color Doppler, a nondilated inferior vena cava, and a small anterior pericardial effusion (0.53 cm) without chamber compromise. Segmental wall motion, global longitudinal strain, and pulmonary artery systolic pressure could not be assessed because of the limited window and the absence of measurable tricuspid regurgitation. N-terminal pro-B-type natriuretic peptide (NT-proBNP) was normal.

The differential diagnosis included malignant serosal involvement, infection (including tuberculosis), heart failure, nephrotic syndrome, venous thromboembolism, hepatic disease, lymphatic obstruction, and immune-mediated serositis. Several features supported probable pembrolizumab-associated immune-mediated polyserositis with an eosinophilic pleural-fluid phenotype: no pleural or pericardial effusion on computed tomography six weeks earlier; a temporal relationship to pembrolizumab; peripheral eosinophilia preceding admission; eosinophilic pleural fluid; negative cytology, cultures, serologies, and proteinuria; preserved biventricular function with normal N-terminal pro-B-type natriuretic peptide; no radiologic tumor progression; and rapid early clinical and biochemical improvement after pembrolizumab withdrawal and corticosteroids. Later imaging suggested possible portal-hypertension physiology, which may have contributed to residual or recurrent fluid accumulation but did not fully explain the acute December 2025 eosinophilic exudative pleural syndrome.

Pembrolizumab was permanently discontinued. The patient received therapeutic thoracentesis, diuretics, and methylprednisolone 1 mg/kg/day followed by a 6-week oral prednisone taper, consistent with guideline-based management of moderate-to-severe immune-related adverse events [4,5,6]. Clinical improvement was evident within days. At the first outpatient review on 30 December 2025, the dyspnea had resolved, and the abdominal distension was improving. Laboratory tests from 24 December 2025 showed the resolution of eosinophilia (0.1%; absolute eosinophil count 0.01 × 10^9^/L), CRP < 1.0 mg/L, creatinine 93 μmol/L, and albumin 35.8 g/L. Follow-up computed tomography on 27 January 2026 showed persistent bilateral pleural effusions and increased ascites but no evidence of recurrent renal cell carcinoma; mediastinal/hilar nodes were stable or smaller, and radiologic signs suggestive of chronic liver disease/portal hypertension were noted. The prednisone taper was completed on 30 January 2026. At review on 3 March 2026, he remained off pembrolizumab and M1-NED, with slowly improving grade 1 dyspnea/ascites and mild periorbital edema; albumin was 35.6 g/L, eosinophils had mildly recurred to 1.0 × 10^9^/L, and NT-proBNP was 32 ng/L. Because the symptoms were not severe, and no new organ dysfunction or radiologic progression was documented, this mild recurrent eosinophilia was managed with clinical and laboratory surveillance.

After the index episode, follow-up interpretation was complicated by a separate April 2026 admission for contained perforated cholecystitis. This later episode resolved clinically without recurrent anasarca and differed from the December presentation. The blood differential was neutrophilic rather than eosinophilic, ascitic fluid was chylous and non-eosinophilic (triglycerides 21.44 mmol/L, eosinophils 1%, serum-ascites albumin gradient (SAAG) 11.0 g/L), and pleural cytology was negative for malignancy. The CT on 17 April 2026 showed a patent porto-spleno-mesenteric axis, recanalization of the umbilical vein/collateral circulation, and near-resolution of the perivesicular abscess. Liver elastography on 21 April 2026 showed liver stiffness 6.5 kPa with reliable acquisition parameters and controlled attenuation parameter 204 dB/m, arguing against advanced fibrosis or significant steatosis. We therefore considered later fluid accumulation multifactorial and clinically distinct from the index eosinophilic pleural syndrome.

## 3. Discussion

### 3.1. Targeted Review Strategy

To contextualize this presentation and inform the practical considerations below, we performed a targeted non-systematic literature review using PubMed. PubMed was searched on 2 April 2026 and rechecked before resubmission using predefined strings combining immune checkpoint inhibitor terms with serositis, pleural effusion, pericardial effusion, ascites, polyserositis, and eosinophilia; the exact search strings are provided in Supplementary Table S2. A predefined eligibility cutoff of 31 January 2026 was used for case-level data extraction to preserve a stable comparison set for Supplementary Table S1. We included case reports and case series describing immune checkpoint inhibitor-associated pleural, pericardial, peritoneal, or multi-compartment serosal inflammation, and we excluded reports in which the effusion was clearly explained by malignant progression, infection, surgery/procedures, or non-ICI causes. Titles and abstracts were screened for relevance, full texts were reviewed when the report appeared to contain a clinically relevant serosal phenotype, and representative case reports/small series were selected to capture the range of phenotypes and reporting practices. Extracted variables included the immune checkpoint inhibitor used, serosal manifestation(s), whether effusion was sampled, whether a serosal fluid differential count was reported, whether an effusion eosinophil percentage was documented, and selected diagnostic and management features. The final extraction set comprised 10 representative reports, detailed in Supplementary Table S1. This review was designed to contextualize fluid-differential reporting and was not designed to estimate incidence or to exclude every possible unpublished or non-indexed case. The completed CARE checklist is provided in Supplementary Table S3.

### 3.2. Immune Checkpoint Inhibitor-Associated Serositis: Clinical Spectrum and Timing

Immune checkpoint inhibitor-associated serositis has been recognized since the mid-2010s, initially through reports of pericardial tamponade and later through broader case series, pharmacovigilance analyses, and systematic reviews [7,8,9,10,11,12,13,14,23,24,25,26,27,28,29]. The literature remains dominated by pericardial manifestations, but pleural effusions, ascites, and generalized edema/polyserositis are increasingly described [12,13,14,26,30,31]. Conservative management of nivolumab-associated pericardial effusion has also been reported [32]. Pleural phenotypes have been reported in metastatic renal cell carcinoma during nivolumab therapy [33], in lung cancer under pembrolizumab or atezolizumab [30,31], and as pleuropericarditis under pembrolizumab [34]. Morbidity can be substantial: in systematic reviews of pericardial cases, tamponade has been reported in approximately 41%, and pericardiocentesis in approximately 68% [8].

The timing is variable. Many reported pericardial events occur within the first months of treatment, with a median onset around four treatment cycles in systematic reviews, but later presentations after prolonged exposure are well documented [8,13]. Our patient developed symptomatic multi-compartment serositis after six cycles of pembrolizumab, which is well within the reported window for immune-related serosal toxicity. Within the published case literature reviewed here, polyserositis has been reported during adjuvant pembrolizumab for melanoma [12]. In our targeted non-systematic review, we did not identify a previously published adjuvant renal cell carcinoma case with the same eosinophilic pleural-fluid phenotype, although this statement should be interpreted within the limitations of the targeted search strategy.

### 3.3. An Underreported Eosinophilic Phenotype

Eosinophilia is a recognized but incompletely characterized feature of immune checkpoint inhibitor toxicity [15,16,17]. In our targeted review, clearly eosinophilic serosal immune-related adverse events were largely limited to biopsy-proven chronic eosinophilic pleuritis after atezolizumab [30] and fulminant eosinophilic myopericarditis after pembrolizumab [35]. The latter confirms that eosinophil-rich cardiac-serosal inflammation can occur during programmed cell death 1 blockade. It is not directly comparable to our case, however, because myocarditis predominated, and serosal fluid eosinophil percentages were not reported [35]. Among the 10 representative published reports (Table 3; full case-level extraction in Supplementary Table S1), six described some serosal fluid differential information, and only one reported an effusion eosinophil percentage, suggesting that eosinophilic serosal phenotypes may be underrecognized rather than truly absent. By contrast, most published immune checkpoint inhibitor-associated serositis/polyserositis reports describe either lymphocytic or non-specific inflammatory phenotypes or do not report serosal fluid differentials at all [12,13,14,26,27,31,32,33,34]. Other directly relevant pleural reports—including nivolumab-associated lymphocyte-mediated pleuritis in metastatic renal cell carcinoma [33], pembrolizumab-associated pleuropericarditis [34], and a later pleural effusion case that reported lymphocytes 90% and neutrophils 10% without an eosinophil percentage [31]—did not provide effusion eosinophil percentages. Our case, with concurrent peripheral eosinophilia and pleural fluid eosinophilia of 20%, supports a probable underrecognized eosinophilic pleural-fluid phenotype within immune checkpoint inhibitor-associated polyserositis and highlights a practical reporting gap in the literature. The clinical relevance of identifying this phenotype is not that it mandates a different first-line treatment in steroid-responsive disease. Rather, it improves inflammatory phenotyping, prompts systematic exclusion of competing causes of eosinophilic pleural effusion, and may become therapeutically relevant if the course becomes steroid-dependent or steroid-refractory.

### 3.4. Mechanistic Considerations

Direct mechanistic data remain limited. In pericardial immune-related adverse events, biopsy and cytology data have most often suggested a T-cell-predominant inflammatory process without tumor involvement [27,28,29]. The coexistence of peripheral eosinophilia and eosinophil-rich pleural fluid in our patient suggests type 2-skewed inflammation, with interleukin-5 as a biologically plausible mediator [15,17,36]. However, because no serosal biopsy or cytokine profiling was available, this mechanistic interpretation should be considered hypothesis-generating rather than definitive. Emerging case experience with mepolizumab or benralizumab for eosinophilic immune-related toxicities is encouraging [36], but evidence in serositis specifically remains sparse. Eosinophil-directed biologic therapy should therefore not be inferred from this case as standard management; it may be considered only in selected steroid-dependent or steroid-refractory eosinophil-rich immune-related toxicity after multidisciplinary discussion.

### 3.5. Diagnostic Approach in Oncology Practice

Immune-mediated serositis remains a diagnosis of exclusion. In patients receiving immune checkpoint inhibitors, the differential diagnosis of new effusions includes malignant serosal involvement, infection (including tuberculosis), heart failure, nephrotic syndrome, liver disease, venous thromboembolism, lymphatic obstruction, and overlapping immune-related toxicities such as myocarditis [4,5,6,18,19,20,21,22,37]. Dedicated pericarditis guidance may also inform the evaluation of pericardial phenotypes [38]. Earlier PD-1 reports also showed that new pleural or pericardial effusions and inflammatory ascites may represent pseudoprogression rather than unequivocal progression, reinforcing the need for serial cytology, imaging correlation, and cautious attribution in patients who otherwise appear to be deriving oncologic benefit [39,40]. Table 4 summarizes a practical diagnostic approach. This case illustrates why pleural fluid differential counts matter: the 20% eosinophil fraction helped define the inflammatory phenotype, while negative cytology, negative microbiology, preserved cardiac function, and rapid steroid response made malignant, infectious, and hemodynamic causes less likely.

The mildly elevated pleural fluid adenosine deaminase could have prompted overdiagnosis of tuberculous pleuritis, but commonly used thresholds are not absolute, and false positives occur in non-tuberculous inflammatory or malignant settings [20]. In an eosinophilic exudate with negative microbiology and no other clinical support for tuberculosis, isolated borderline adenosine deaminase elevation should therefore be interpreted cautiously [20,21]. Conversely, eosinophilia does not exclude malignancy [21]. For clinicians managing immunotherapy toxicity, these findings underscore that “exudative effusion” or “negative cytology” alone are insufficient. Differential cell counts, microbiologic studies, and careful oncologic reassessment remain essential.

### 3.6. Management and Rechallenge in the Adjuvant Setting

Management depends on the symptom burden, compartment involvement, and suspected mechanism (Table 5). Symptomatic moderate-to-large effusions generally require diagnostic and therapeutic drainage, temporary interruption of immune checkpoint inhibitor therapy, and grade-based systemic corticosteroids once infection has been reasonably excluded [4,5,6]. In hemodynamically significant pericardial disease, urgent drainage is mandatory, and concomitant myocarditis must be assessed [4,5,6,37].

Steroid-dependent or steroid-refractory pericardial phenotypes may require multidisciplinary input [41,42]. In selected situations, steroid-sparing immunomodulation has been reported [36,42]. Rechallenge after immune-related serositis has been reported, particularly after low-grade or fully resolved pericardial phenotypes, but the recurrence risk is uncertain [25,43]. Importantly, the available rechallenge experience derives mainly from non-eosinophilic pericardial phenotypes. Comparable rechallenge data after clearly eosinophilic pleuritis or eosinophilic cardiac-serosal disease are lacking; so, extrapolation to eosinophilic presentations should be cautious. The adjuvant setting deserves particular caution: unlike metastatic disease, treatment is given to reduce recurrence risk rather than to control measurable tumor burden. In our patient, the permanent discontinuation of pembrolizumab after multi-compartment probable immune-mediated serositis and rapid initiation of corticosteroids was clinically appropriate.

### 3.7. Practical Implications and Limitations

This case also has reporting implications. The combination of anasarca, multi-compartment effusions, peripheral eosinophilia, and eosinophilic pleural fluid suggests that some published cases labeled simply as “polyserositis” or “generalized edema” may have shared an eosinophilic inflammatory phenotype that went unrecognized, because cell differentials were not documented [12,14]. Routine reporting of serosal fluid differential counts, including eosinophil percentages, would make future case reports more clinically informative. Such reporting does not imply that eosinophil-directed therapy is required in steroid-responsive disease, but it may help define a subgroup for future study and for multidisciplinary consideration if steroid-dependent or steroid-refractory eosinophil-rich toxicity occurs.

Strengths of this report include the careful exclusion of malignant and infectious causes, explicit serosal fluid phenotyping, an expanded clinical and laboratory timeline, and a targeted review focused on a clinically actionable reporting gap. Limitations include the single-patient design, absence of serosal biopsy or cytokine profiling, and the fact that the adjuvant treatment decision should not be read as a direct representation of the KEYNOTE-564 M1-NED subgroup, because the resected metastasis was osseous, and the nephrectomy-to-metastasectomy interval was slightly more than one year. The case also cannot fully disentangle the contribution of possible non-cirrhotic portal-hypertension physiology, lymphatic dysfunction, or other systemic factors to residual or recurrent late fluid accumulation. The later April 2026 episode occurred during contained perforated cholecystitis and produced chylous non-eosinophilic ascites/chylothorax that resolved clinically. It was therefore treated as a separate confounder and not as recurrent eosinophilic immune-mediated serositis. The December index presentation, however, is more confidently interpreted as probable pembrolizumab-associated eosinophilic polyserositis, because eosinophilia preceded overt serositis, the pleural fluid was eosinophilic, competing infectious, malignant, cardiac, renal, and advanced cirrhotic causes were not supported, and the symptoms and laboratory abnormalities improved promptly after drug withdrawal and corticosteroids. The case should therefore be interpreted as clinically instructive and hypothesis-generating.

## 4. Conclusions

Pembrolizumab-associated polyserositis with eosinophilic pleural effusion is a rare but clinically important immune-related adverse event that can mimic infection, cancer recurrence, or systemic fluid-retention states during adjuvant pembrolizumab. In patients with new serosal effusions under immune checkpoint blockade, routine differential cell counts in drained fluid may reveal an eosinophilic pleural-fluid phenotype that would otherwise be missed. Early recognition, systematic exclusion of competing etiologies, and prompt corticosteroid-based management can lead to rapid clinical improvement. This is especially relevant in the adjuvant setting, including M1-NED scenarios in which trial eligibility details should be documented carefully, because distinguishing toxicity from relapse directly influences treatment interruption and follow-up strategy.

## Figures and Tables

**Figure 1:**
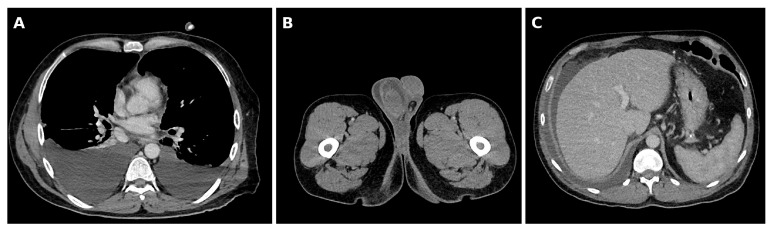
Contrast-enhanced CT showing large bilateral pleural effusions (**A**), marked scrotal and soft-tissue edema (**B**), and mild ascites (**C**).

**Table 1:** Expanded clinical and laboratory timeline relative to pembrolizumab initiation and follow-up.

Timepoint	Event
21 May 2024	Right nephrectomy and adrenalectomy for clear cell renal cell carcinoma (pT1b, grade 2).
30 May 2025	Complete resection of solitary left proximal humerus osseous metastasis with negative margins.
3 July 2025	Restaging CT showed no evidence of disease; metastatic disease with no evidence of disease (M1-NED) achieved.
15 July 2025	Pembrolizumab 200 mg every 3 weeks initiated using the KEYNOTE-564 dose and schedule 46 days after metastasectomy and 12 days after restaging CT. The nephrectomy-to-metastasectomy interval was 374 days (12 months and 9 days). Because the metastasis was osseous, and the interval slightly exceeded one year, the case did not fully match the published trial-defined M1-NED subgroup.
24 October 2025 (after cycle 5)	Staging CT showed mediastinal/axillary adenopathy but no pleural or pericardial effusion.
18 November 2025 (pre-cycle 7 visit)	Pembrolizumab held after a local diagnosis of presumed acute orchitis; peripheral eosinophilia already present (1.30 × 10^9^/L; 15.8%). In retrospect, the scrotal symptoms were considered more consistent with early edema/anasarca than proven isolated infectious orchitis.
29 November 2025	Outside CT: new mild ascites and bilateral pleural effusions with hepatomegaly/steatosis.
9 December 2025	Progressive anasarca despite furosemide; creatinine 126 μmol/L, albumin 32.8 g/L; admission planned.
15 December 2025	Thoracentesis (1.5 L) yielded an exudative pleural effusion by Light’s criteria (protein criterion met), with 20% eosinophils, ADA 42.8 U/L, negative cytology, and negative bacterial, mycobacterial, and fungal studies.
16 December 2025	Technically limited transthoracic echocardiography showed LVEF 71%, visually preserved RV function, nondilated IVC, and a small anterior pericardial effusion (0.53 cm) without chamber compromise.
18 December 2025	Discharged on 6-week prednisone taper; pembrolizumab permanently discontinued.
30 December 2025 (~2 weeks after discharge)	First outpatient review: no dyspnea, improving abdominal distension; laboratory tests from 24 December 2025 showed eosinophils 0.1% (0.01 × 10^9^/L), CRP < 1.0 mg/L, creatinine 93 μmol/L, and albumin 35.8 g/L.
27 January 2026 (~6 weeks)	CT: persistent bilateral pleural effusions and increased ascites but no oncologic progression; mediastinal/hilar nodes stable or smaller; radiologic chronic liver disease/portal-hypertension changes described.
3 March 2026 (~11 weeks)	After completion of prednisone taper on 30 January 2026: grade 1 residual dyspnea/ascites and mild periorbital edema; albumin 35.6 g/L, eosinophils 1.0 × 10^9^/L, NT-proBNP 32 ng/L; still M1-NED.
April 2026 (separate later admission)	Contained perforated cholecystitis with febrile inflammation and chylous non-eosinophilic ascites/chylothorax. Ascitic triglycerides were 21.44 mmol/L, eosinophils 1%, and serum-ascites albumin gradient (SAAG) 11.0 g/L. Liver elastography showed liver stiffness 6.5 kPa and CAP 204 dB/m. This separate episode resolved clinically without recurrent anasarca and was considered distinct from the December eosinophilic pleural syndrome.

Abbreviations: ADA, adenosine deaminase; CAP, controlled attenuation parameter; CRP, C-reactive protein; CT, computed tomography; IVC, inferior vena cava; LVEF, left ventricular ejection fraction; M1-NED, metastatic disease with no evidence of disease; NT-proBNP, N-terminal pro-B-type natriuretic peptide; RV, right ventricular; SAAG, serum-ascites albumin gradient.

**Table 2:** Pleural fluid and paired serum findings from the index thoracentesis on 15 December 2025.

Parameter	Pleural Fluid	Paired Serum	Interpretation
Appearance	Serous	-	Therapeutic thoracentesis, 1.5 L
Total nucleated cells	1020/mm^3^	-	Cellular inflammatory effusion
RBC	1000/mm^3^	-	Minimal blood contamination
Lymphocytes	39%	2.93 × 10^9^/L	Lymphocyte-rich component
Monocytes/macrophages	31%	1.27 × 10^9^/L monocytes	Inflammatory component
Mesothelial cells	8%	-	Reactive mesothelial cells
Eosinophils	20%	1.27 × 10^9^/L	Eosinophilic pleural effusion
Basophils	1%	0.15 × 10^9^/L	Minor component
Protein	35.3 g/L	58.8 g/L	Pleural/serum protein ratio 0.60; exudate by Light’s criteria (protein criterion met)
LDH	138 U/L	269 U/L	Pleural/serum LDH ratio 0.51; LDH criterion not met
Glucose	5.6 mmol/L	Not available	Preserved pleural glucose
pH	Not available	-	Not performed/not available
ADA	42.8 U/L	-	Borderline elevation
Cytology	Reactive mesothelial/inflammatory cells; no malignant cells	-	Negative for malignancy
Bacterial culture	Negative	-	Final negative
AFB smear/mycobacterial culture	Negative	-	Final negative; culture finalized 4 February 2026
Fungal culture	Negative	-	Final negative

Abbreviations: ADA, adenosine deaminase; AFB, acid-fast bacilli; LDH, lactate dehydrogenase; RBC, red blood cells.

**Table 3:** Summary of serosal-fluid reporting features across 10 representative published immune checkpoint inhibitor-associated serositis reports. Full case-level extraction is provided in Supplementary Table S1.

Reporting Feature Among 10 Representative Reports	*n*/10
Effusion sampled	6/10
Effusion not sampled	2/10
Sampling not specified/reported	2/10
Any serosal-fluid differential reported, including partial reporting	6/10
Complete or clinically interpretable differential reported	3/10
Effusion eosinophil percentage reported	1/10

**Table 4:** Suggested diagnostic approach to suspected immune checkpoint inhibitor-associated serositis, synthesized from the current irAE guidance, pleural-effusion literature, cardio-oncology guidance, and selected pseudoprogression comparator reports [4,5,6,18,19,20,21,22,37,38,39,40].

Domain	Key Elements	Rationale
Oncologic assessment	CT/PET-CT for tumor response; exclude new serosal nodularity; repeat sampling or tissue biopsy if uncertainty persists; tumor markers if relevant	Differentiate irAE from malignant serosal involvement or progression
Serosal fluid analysis	Cell count with differential (include eosinophil percentage); protein and LDH (Light’s criteria); glucose; pH when available; triglycerides/cholesterol if chylous effusion is possible; cytology; flow cytometry, if lymphoma is possible	Characterize inflammatory phenotype; detect malignancy; identify eosinophilic or chylous effusion
Microbiology	Gram stain/culture; fungal culture; AFB smear/culture; TB NAAT, ADA, and IGRA as appropriate	Exclude infection before immunosuppression
Cardiopulmonary evaluation	ECG; troponin; natriuretic peptides; echocardiography; consider CMR, if myocarditis is suspected; drainage, if tamponade or diagnostic uncertainty is present	Assess pericardial involvement and rule out myocarditis or heart failure
Systemic causes	Renal function; urinalysis/proteinuria; liver tests; albumin; INR/platelets; portal/hepatic venous assessment when indicated; thyroid function; venous duplex or CTPA, if thromboembolism is suspected	Exclude cardiac, renal, hepatic/portal-hypertensive, endocrine, or thromboembolic causes of effusions and edema
Eosinophil context	Serial absolute eosinophil counts; review for rash, asthma, atopy; basic autoimmunity, if indicated	Characterize eosinophilic phenotype and consider alternative inflammatory etiologies

Abbreviations: ADA, adenosine deaminase; AFB, acid-fast bacilli; CMR, cardiac magnetic resonance; CTPA, computed tomography pulmonary angiography; ECG, electrocardiography; IGRA, interferon-gamma release assay; irAE, immune-related adverse event; LDH, lactate dehydrogenase; NAAT, nucleic acid amplification test; PET-CT, positron emission tomography–computed tomography; TB, tuberculosis.

**Table 5:** Practical management of immune checkpoint inhibitor-associated serositis, synthesized from the current irAE/pericardial guidance and selected reported steroid-dependent, steroid-sparing, and rechallenge cases [4,5,6,36,37,38,41,42,43].

Scenario	Management Considerations
Asymptomatic small effusion	Repeat imaging; evaluate for progression and infection; consider holding immune checkpoint inhibitor therapy, if rapid accumulation occurs; multidisciplinary consultation as needed
Symptomatic moderate-to-large effusion	Diagnostic and therapeutic drainage with fluid differential and cytology; hold immune checkpoint inhibitor therapy; systemic corticosteroids (typically prednisone 0.5–1 mg/kg/day for moderate or 1–2 mg/kg/day for severe presentations) once infection is excluded; slow taper
Pericardial effusion with hemodynamic compromise	Urgent pericardiocentesis or surgical window; intensive care monitoring; high-dose steroids once infection is excluded; evaluate for myocarditis (troponin, CMR)
Steroid-refractory or steroid-dependent disease	Multidisciplinary input; steroid-sparing agents such as azathioprine or infliximab have been reported for selected pericardial phenotypes; interleukin-5 axis blockade (mepolizumab, benralizumab) is hypothesis-generating for selected steroid-dependent or steroid-refractory eosinophil-driven immune-related toxicity and is not standard first-line management; interleukin-1 blockade may be considered for recurrent pericarditis phenotypes when evidence and expertise support its use
Immune checkpoint inhibitor rechallenge	Individualize according to severity, compartment, inflammatory phenotype, and oncologic context; consider only after complete resolution with close monitoring; generally avoid after life-threatening, multi-compartment, or eosinophil-rich presentations in the adjuvant setting unless there is a compelling oncologic rationale

Abbreviation: CMR, cardiac magnetic resonance.

## Data Availability

All data generated or analyzed during this study are included in this article. Additional de-identified details are available from the corresponding author on reasonable request, subject to privacy restrictions.

## Supplementary Materials

The following supporting information can be downloaded at https://www.mdpi.com/article/10.3390/curroncol33060314/s1, Table S1: Case-level data extraction from 10 representative published immune checkpoint inhibitor-associated serositis reports; Table S2: Targeted review search strategy, screening approach, inclusion/exclusion criteria, and rationale for representative case selection; Table S3: Completed CARE checklist. This case report was prepared in accordance with the CARE reporting guideline [44].

## Abbreviations

The following abbreviations are used in this manuscript:

ADA	adenosine deaminase
AFB	acid-fast bacilli
CAP	controlled attenuation parameter
CMR	cardiac magnetic resonance
CRP	C-reactive protein
CT	computed tomography
CTPA	computed tomography pulmonary angiography
ECG	electrocardiography
ICI	immune checkpoint inhibitor
IGRA	interferon-gamma release assay
irAE	immune-related adverse event
IVC	inferior vena cava
LDH	lactate dehydrogenase
LVEF	left ventricular ejection fraction
M1-NED	metastatic disease with no evidence of disease
NAAT	nucleic acid amplification test
NT-proBNP	N-terminal pro-B-type natriuretic peptide
PET-CT	positron emission tomography-computed tomography
RBC	red blood cells
RV	right ventricle/right ventricular
SAAG	serum-ascites albumin gradient
TB	tuberculosis
