# Supplementary material for: Pembrolizumab-Associated Polyserositis with Eosinophilic Pleural Effusion During Adjuvant Therapy for Clear Cell Renal Cell Carcinoma: A Case Report and Targeted Review
Source: Curr Oncol. 2026 May 27;33(6):314. doi: 10.3390/curroncol33060314 (PMC13298109; doi:10.3390/curroncol33060314)
Supplement: Supplementary file 1 [file curroncol-33-00314-s001.zip › curroncol-4315616-supplementary.pdf]

## Supplementary Materials

Supplementary Table S1. Case-level data extraction from 10 representative published immune checkpoint inhibitor-associated serositis reports

This supplementary table expands the variables summarized in Table 3 of the main manuscript and makes the reporting-gap claim explicit.

| Author/year [ref.] | ICI agent | Serosal manifestation(s) | Effusion sampled | Any serosal fluid differential reported? | Effusion eosinophil % reported? | Key note relevant to interpretation |
|---|---|---|---|---|---|---|
| Verhaert 2023 [41] | Pembrolizumab | Pericarditis (steroid-dependent) | Not specified | No | No | Steroid-dependent pericardial phenotype; no fluid differential data available |
| Ali 2022 [25] | Nivolumab | Recurrent pericarditis | No | N/A | No | Rechallenge-focused recurrent pericarditis report; no sampled effusion differential |
| Saade 2019 [27] | Nivolumab | Pericardial effusion/tamponade + literature review | Yes | Partially | No | Pericardial cases/review; cytology or leukocyte data reported, but no eosinophil percentage |
| Shaheen 2018 [32] | Nivolumab | Pericardial effusion | No | N/A | No | Conservative corticosteroid management report; no fluid differential data available |
| Castelli 2024 [26] | Nivolumab | Polyserositis/chylous ascites | Yes | Partially | No | Polyserositis case with lymphocyte-predominant/chylous features; no eosinophil percentage |
| Sawada 2021 [13] | Nivolumab | Pleural + pericardial effusions | Yes | Partially | No | Late pleuropericardial irAE after prolonged exposure; pleural fluid note of elevated lymphocyte count, but no full differential or eosinophil percentage |
| Suarez 2023 [34] | Pembrolizumab | Pleuropericarditis | Not reported | No | No | Pleuropericarditis comparator; no sampled differential reported |
| Yanagihara | Nivolumab | Lymphocyte- | Yes | Yes | No | Metastatic RCC |

| | | | | | | |
|---|---|---|---|---|---|---|
| 2017 [33] | | mediated pleuritis (RCC) | | | | pleural comparator; lymphocyte-mediated pleuritis without eosinophil percentage |
| Shen 2021 [31] | Pembrolizumab | Progressive pleural effusion | Yes | Yes | No | Pleural effusion comparator with lymphocytes 90% and neutrophils 10%; no eosinophil percentage |
| Lin 2021 [30] | Atezolizumab | Chronic pleuritis/recurrent pleural effusion | Yes | Yes | Yes (13%) | Only representative report documenting an effusion eosinophil percentage |

Abbreviations: ICI, immune checkpoint inhibitor; irAE, immune-related adverse event; N/A, not applicable; RCC, renal cell carcinoma.

Supplementary Table S2. Targeted review search strategy, screening approach, inclusion/exclusion criteria, and rationale for representative case selection

| Element | Description |
|---|---|
| Database and dates | PubMed was searched on 2 April 2026 and rechecked before resubmission. A predefined case-level extraction cutoff of 31 January 2026 was used for the representative extraction set. |
| Search string 1 | ("immune checkpoint inhibitor" OR pembrolizumab OR nivolumab OR atezolizumab) AND (serositis OR polyserositis OR "generalized oedema" OR edema) |
| Search string 2 | ("immune checkpoint inhibitor" OR pembrolizumab OR nivolumab OR atezolizumab) AND ("pleural effusion" OR pleuritis OR pleurisy) |
| Search string 3 | ("immune checkpoint inhibitor" OR pembrolizumab OR nivolumab OR atezolizumab) AND ("pericardial effusion" OR pericarditis OR tamponade) |
| Search string 4 | (pembrolizumab OR nivolumab OR atezolizumab OR "immune checkpoint inhibitor") AND (eosinophilia OR eosinophilic) AND (pleural OR pericardial OR serositis) |
| Search string 5 | ("renal cell carcinoma" OR RCC) AND (nivolumab OR pembrolizumab OR "immune checkpoint inhibitor") AND (pleuritis OR "pleural effusion" OR serositis OR polyserositis) |
| Inclusion criteria | Case reports or case series describing immune checkpoint inhibitor-associated pleural, pericardial, peritoneal, generalized edema, or multi-compartment serosal inflammatory phenotypes. Reports were prioritized if they included fluid sampling, cytology, differential cell counts, diagnostic exclusion, or management details. |
| Exclusion criteria | Reviews without extractable case-level data, non-serosal eosinophilia without pleural/pericardial/peritoneal involvement, effusions clearly attributable to malignant progression or infection, non-ICI-related effusions, and reports without sufficient clinical detail for the variables |

| | |
|---|---|
| | extracted in Supplementary Table S1. |
| Screening process | Titles and abstracts were screened for relevance. Full texts were reviewed when the title/abstract suggested an ICI-associated serosal phenotype or when the paper was a review likely to identify additional cases. Reference lists of relevant reviews/cases were checked for additional comparator reports. |
| Rationale for 10 representative reports | The extraction set was selected to represent clinically relevant serosal phenotypes and reporting practices, including pericardial disease, pleural disease, polyserositis/chylous ascites, RCC comparators, pembrolizumab comparators, and eosinophil-rich pleural disease. The review was targeted, not systematic, and was not designed to estimate incidence. |

Supplementary Table S3. Completed CARE checklist

| CARE item | Reported in revised manuscript | Comment |
|---|---|---|
| Title | Title | Identifies the case report topic, intervention, phenotype, and cancer context. |
| Keywords | Keywords | Includes pembrolizumab, renal cell carcinoma, polyserositis, eosinophilic pleural effusion, and case report. |
| Abstract | Abstract | Summarizes rationale, clinical presentation, diagnostic findings, intervention, outcome, and main lesson. |
| Introduction | Introduction | Provides background on adjuvant pembrolizumab, ICI serositis, and eosinophilic effusion phenotype. |
| Patient information | Detailed Case Description | Age, oncologic history, treatment context, and relevant comorbidities are reported in de-identified form. |
| Clinical findings | Detailed Case Description and Figure 1 | Reports anasarca, pleural effusions, ascites, scrotal/facial edema, eosinophilia, and pericardial effusion. |
| Timeline | Table 1 | Expanded clinical and laboratory timeline is provided. |
| Diagnostic assessment | Detailed Case Description; Table 2; Table 4 | Includes pleural-fluid profile, cytology, microbiology, cardiac assessment, renal/hepatic evaluation, and differential diagnosis. |
| Therapeutic intervention | Detailed Case Description; Table 5 | Reports pembrolizumab discontinuation, thoracentesis, corticosteroids, diuretics, and management rationale. |
| Follow-up and outcomes | Detailed Case Description; Table 1 | Reports clinical/laboratory response, recurrent mild eosinophilia, later confounded chylous/infectious episode, and oncologic follow-up. |
| Discussion | Discussion | Discusses diagnostic reasoning, literature context, mechanisms, management, and limitations. |
| Patient perspective | Not available | A formal patient-perspective statement |

| | | |
|---|---|---|
| | | was not obtained. Written consent for publication was obtained. |
| Informed consent | Informed Consent Statement | Written informed consent was obtained for publication of the case and de-identified data. |
